# Assay of Antioxidant Potential of Two Filamentous Fungi Isolated from the Indonesian Fermented Dried Cassava

**DOI:** 10.3390/antiox5010006

**Published:** 2016-02-02

**Authors:** Sugiharto Sugiharto, Turrini Yudiarti, Isroli Isroli

**Affiliations:** Faculty of Animal and Agricultural Sciences, Diponegoro University, Semarang 50275, Indonesia; tyudiarti@yahoo.co.id (T.Y.); isroliundip02@yahoo.com (I.I.)

**Keywords:** antioxidant potential, antioxidant constituents, *Acremonium charticola*, *Rhizopus oryzae*, *gathot*

## Abstract

The antioxidant capacity and antioxidant constituents of two filamentous fungi (*Acremonium charticola* and *Rhizopus oryzae*) isolated from the Indonesian fermented dried cassava (*gathot*) were evaluated in the present study. The antioxidant capacity of the fungal crude extracts was assessed based on the 2,2′-azino-bis(3-ethyl-benzthiazolin-6-sulfonicacid) (ABTS) method. Total phenolics were determined based on the Folin-Ciocalteu method, while the flavonoids content in the fungal extracts was determined by the spectrophotometric method with aluminum chloride. Total tannins were estimated by the Folin-Denis method. The ABTS^+^ radical scavenging activity was higher (*p* < 0.01) in *A. charticola* compared to that in *R. oryzae* and ascorbic acid (as a control). A higher (*p* < 0.01) content of total phenolics was detected in *A. charticola* than that in *R. oryzae*. Total flavonoids were higher (*p* < 0.01) in *R. oryzae* as compared with that in *A. charticola*. The fungus *A. charticola* had a higher (*p* < 0.01) level of total tannins than *R. oryzae*. In conclusion, both filamentous fungi isolated from the Indonesian fermented dried cassava exhibited antioxidant potentials as indicated by their capabilities to scavenge ABTS^+^. *A. charticola* had a higher antioxidant capacity than *R. oryzae*. The antioxidant capacity of *A. charticola* was attributed mainly to its phenolics and tannins contents.

## 1. Introduction

Reactive oxygen species (ROS) are products of normal cellular metabolism from living organisms. At low to moderate concentrations, these chemically reactive molecules beneficially affect metabolic, immune defense and cellular processes, but at high concentrations they can promote pathological conditions in the body [1]. The endogenous antioxidant system plays a crucial role in controlling ROS levels and maintaining the balances of the oxidation-reduction (redox) status of the body. However, when the endogenous antioxidants fail to counteract the excessive production of ROS, the exogenous antioxidants would be useful in synchronizing the redox balance status [2]. Dietary sources, such as plants, herbs, spices and herbal extracts, have been recognized as good sources of natural antioxidants. These food ingredients contain phenolic compounds that can act as antioxidants and thus exert protective health effects [3].

It is generally known that fermentation can increase the antioxidant activity of foods or feeds [4]. Schmidt *et al.* [5] reported that fermentation of rice bran with the *Rhizopus oryzae* fungus increased the free phenolics content by more than 100%, and thus increased the antioxidant capacity of the rice bran. In Indonesia, the knowledge of fermentation has long been implemented to produce *gathot*, which is a traditional fermented dried cassava [6]. A previous study by Purwandari *et al.* [7] revealed that *gathot* flour had high antioxidant activity, which was indicated by the 2,2-diphenylpicrylhydrazyl (DPPH) scavenging ability of 90.33% and total phenolic compounds of 419.43 mg/100 g. To date, the definite mechanisms by which fermentation increases the antioxidant capacity and total phenolic compounds in *gathot* remain unknown. Microbial hydrolysis reaction or the structural breakdown of plant cell walls leading to the liberation or synthesis of various antioxidant compounds during fermentation could be one possible mechanism [4].

Studies demonstrated that filamentous fungi *Aspergillus* PR78, *Aspergillus* PR66 [8] and *Monascus purpureus* [9] are good sources of phenolic compounds. With this background, and given that *gathot* is made through spontaneous fungal fermentation [6], the fungi involved in the production of *gathot* probably contribute to the antioxidant capacity of *gathot* in part through the production of phenolic compounds or other compounds associated with antioxidant activity. However, comprehensive descriptions of the antioxidant capacity and the production of phenolic compounds by these fungi have remained unelucidated prior to this study. In our previous study, we isolated two filamentous fungi from *gathot*, *i.e.*, *Acremonium charticola* and *Rhizopus oryzae* [6]. Hence, the aim of the present study was to assess the antioxidant potential and antioxidant constituents of these two filamentous fungi isolated from *gathot*.

## 2. Materials and Methods

### 2.1. Preparation of Fungal Crude Extracts

Fungal isolates of *A. charticola* and *R. oryzae* were obtained from our own culture collection [6]. Fungi were inoculated onto potato dextrose broth in conical flask (250 mL, Pyrex, Tokyo, Japan), and after incubation for three days at 37 °C the fungal colonies were observed to ensure the purity of the culture. The grown cultures were then transferred into conical tubes (50 mL, Thermo Fisher Scientific, Rochester, NY, USA). Extraction was initiated by centrifuging the cultures at 5000 rpm for 10 min. The filtrate (1 g) was dissolved in 100 mL methanol, ultrasonicated for 30 min and macerated for three days. During the maceration period, homogenization was performed each day using magnetic stirrer (Thermo Fisher Scientific) for 30 min at room temperature. The homogenate was subsequently evaporated with rotary vacuum evaporator (50 °C, 100 rpm, Sigma-Aldrich, St. Louis, MO, USA) until the volume became 25 mL.

### 2.2. Determination of Antioxidant Activity

Antioxidant capacities of the fungal extracts were determined based on the 2,2′-azino-bis(3-ethyl-benzthiazolin-6-sulfonicacid) (ABTS) method according to Sun *et al.* [10] with few modifications. To form ABTS^+^, potassium persulfate (K_2_S_2_O_8_, Merck KGaA, Darmstadt, Germany) was added to ABTS, mixed and kept in the dark for 16 h at room temperature. Phosphate buffer (10 mM, pH 7.4, Merck KGaA) was used to dilute ABTS^+^ stock solution to a final absorbance of *ca.* 0.7 at 734 nm. Fungal extracts or ascorbic acid (Sigma-Aldrich) used as standard (50 µL) were dissolved in 3 mL of diluted ABTS^+^ solution. The scavenging activity of the fungal extracts was assessed from the percentage of decolorization at 734 nm after 2 min of reaction at room temperature. The ABTS^+^ scavenging activity (%) was calculated using the equation as follows: ((OD_734control_ − OD_734sample_)/OD_734control_) × 100%. The assay was conducted in triplicate.

### 2.3. Determination of Total Phenolics Content

Determination of total phenolics content in the fungal extracts was performed based on Folin-Ciocalteu method according to Orak [11] with few modifications. Fungal homogenate (0.5 mL), distilled water (8 mL), Folin-Ciocalteu reagent (0.5 mL, Merck KGaA) and sodium carbonate (Na_2_CO_3_, 1 mL, Merck KGaA) were mixed. The mixture was incubated at room temperature for 30 min. The absorbance of the solution was subsequently measured at 765 nm with a spectrophotometer. A standard curve was plotted using gallic acid. The assay was conducted in triplicate.

### 2.4. Determination of Total Flavonoids Content

Assay for the content of flavonoids in the fungal extracts was performed by spectrophotometric method with aluminum chloride according to Ghasemzadeh *et al.* [12]. In brief, fungal homogenate (1 mL), 5% sodium nitrite (NaNO_2_, 0.7 mL, Merck KGaA) and 30% ethanol (10 mL) were mixed for 5 min, and then 10% aluminum chloride (AlCl_3_, 0.7 mL, Merck KGaA) was added and mixed altogether. After 6 min, 1 mol/L sodium hydroxide (NaOH, 5 mL, Merck KGaA) was added, the mixture was diluted to 25 mL with 30% ethanol and let to stand for 10 min. The absorbance of the solution was then measured at 430 nm with a spectrophotometer (Shimadzu, Kyoto, Japan). A standard curve was plotted using quercetin. The assay was performed in triplicate.

### 2.5. Determination of Total Tannins Content

Tannins content of the fungal extracts was determined colorimetrically using Folin-Denis reagent according to Chanwitheesuk *et al.* [13]. In brief, fungal homogenate (0.5 mL) was added with 8 mL distilled water, 0.5 mL Folin-Denis reagent (Merck KGaA) and 1 mL sodium carbonate (Na_2_CO_3_, Merck KGaA)_._ The solution was mixed and incubated at room temperature for 30 min. The absorbance of the solution was measured at 760 nm using tannic acid solution (Sigma-Aldrich) as a standard solution. The assay was conducted in triplicate.

### 2.6. Statistical Analysis

Data obtained from ABTS assay were analyzed by analysis of variance (ANOVA) followed by Duncan’s *post hoc* test to assess the difference between mean values. To compare group means of total phenolics, flavonoids and tannins in the fungal extracts, *t*-test was conducted. A significant difference among groups was considered when *p* < 0.05.

## 3. Results

### 3.1. Antioxidant Potential of Fungal Extracts

The antioxidant capacity (ABTS^+^ radical scavenging activity) of the fungal isolates is presented in Table 1. The ABTS^+^ radical scavenging activity differed (*p* < 0.01) between the two fungal isolates and ascorbic acid as a control. The fungus *A. charticola* had stronger antioxidant activity when compared with *R. oryzae* and ascorbic acid.

**Table 1:** Antioxidant activity, total phenolics, flavonoids and tannins contents in two filamentous fungi isolated from *gathot* ^‡^.

Samples	ABTS^+^ Scavenging Activity (%)	Total Phenolics (mg/100 g)	Total Flavonoids (mg/100 g)	Total Tannins (mg/100 g)
*A. charticola*	93.11 ± 0.03 ^a^	26.25 ± 0.39 ^a^	2.92 ± 0.15 ^a^	26.42 ± 0.63 ^a^
*R. oryzae*	14.20 ± 0.01 ^b^	16.08 ± 0.16 ^b^	10.87 ± 0.37 ^b^	16.10 ± 0.20 ^b^
Ascorbic acid	77.63 ± 0.01 ^c^			

^a,b,c^ Values with different letters within the same column were significantly different (*p* < 0.01). ^‡^ Values are mean ± standard deviation.

### 3.2. Antioxidant Constituents of Fungal Extracts

The antioxidant constituents including total phenolics, flavonoids and tannins in the fungal extracts are presented in Table 1. A higher (*p* < 0.01) content of total phenolics was detected in *A. charticola* than that in *R. oryzae*. Total flavonoids were higher (*p* < 0.01) in *R. oryzae* as compared with that in *A. charticola*. The fungus *A. charticola* had a higher (*p* < 0.01) level of total tannins than *R. oryzae*.

## 4. Discussion

The antioxidant capacity and antioxidant constituents of the two filamentous fungi isolated from the Indonesian fermented dried cassava were assessed in the present study. Results showed that both *A. charticola* and *R. oryzae* had antioxidant potentials, as indicated by their capabilities to scavenge ABTS^+^. This finding was consistent with that obtained from our preliminary study [14]. Based on the DPPH assay, it was apparent that both fungal isolates exhibited strong antioxidant potential as indicated by the values of IC_50_ below 100 µg/mL (*i.e.*, the IC_50_ values of *A. charticola*, *R. oryzae* and ascorbic acid were 51.96 ± 0.01, 55.89 ± 0.63 and 1.89 ± 2.88 µg/mL, respectively). In accordance with the present result, the antioxidant capacity of *A. charticola* was significantly higher than that of *R. oryzae* in our earlier study. However, in contrast to the current data, DPPH assay showed that *A. charticola* (as well as *R. oryzae*) had lower antioxidant activities as compared to the ascorbic acid which was used as a standard antioxidant [14]. Our studies therefore further confirm that although ABTS and DPPH assays employ the same reaction mechanisms (*i.e.*, electron transfer assays), the antioxidant data produced by these two methods were not always linear between one another, as also reported by Fidrianny *et al.* [15]. With regard to ascorbic acid, this acid has commonly been used as a standard antioxidant in ABTS and DPPH assays, considering that ascorbic acid is one of the most powerful natural antioxidants and prominent representatives of dietary antioxidants for human [16]. Taken together, our results suggest that apart from the actions of filamentous fungi to liberate antioxidant compounds from plant cell walls [4], fungi themselves could be sources of compounds associated with antioxidant activity.

Several studies have revealed that the antioxidant capacity in filamentous fungi was attributed mainly to their phenolics content [8,9]. As the total phenolics increase, the antioxidant potential of the filamentous fungi increases [8]. In accordance with this, the higher content of total phenolics in *A. charticola* was associated with the higher percentage of inhibition of ABTS^+^, when compared with that in *R. oryzae*. With respect to the levels of phenolic compounds in our fungal extracts, they seemed, however, to be lower compared to that of oranges, which are an eminent source of phenolic compounds in human diets. Note that oranges contain nearly 151 mg gallic acid equivalents (GAE)/100 g fresh mass [17]. Tannin compounds have been reported to contribute to the antioxidant activity of filamentous fungi [9]. In this study, the total tannins were found to be higher in *A. charticola* than in *R. oryzae*. This is consistent with the higher antioxidant potential of *A. charticola* than that of *R. oryzae*. In the human diet, the contribution of tannins in tea (or herbal tea) to the total tannins intake is of particular importance. Tshivhandekano *et al.* [18] reported that tea contains tannins of 0.34 mg/100 g. Thus, compared to the tannins content in tea, the total tannins in our fungal extracts was noticeably higher.

Flavonoids are the largest family of phenolic compounds that are ubiquitously found in human diets. Among the diets, oranges are a significant source of flavonoids as these fruits contain flavonoids of around 69.85 mg/100 g [19]. Indeed, this flavonoids content was significantly higher than that in our fungal extracts. It has been recognized that the presence of flavonoids is associated with the antioxidant potential of foods [3]. In contrast to the latter review, Anjum *et al.* [20] reported that flavonoids exhibited a weak correlation with antioxidant activity in *Cuscuta* sp. extracts. Similarly, Bakhari *et al.* [21] noticed a lacking correlation between total flavonoids and antioxidant activity in *Pereskia bleo* (Kunth). The lacking correlation between total flavonoids and antioxidant potential was also observed in the filamentous fungus *Monascus purpureus* as reported by Smith *et al.* [9]. In accordance with these earlier reports, we did not find any correlation between total flavonoids content and antioxidant capacity in the present fungal extracts, in that the higher content of total flavonoids in *R. oryzae* was not associated with the higher antioxidant capacity in the fungus when compared with *A. charticola*. Conversely, the lower flavonoids content in *A. charticola* was accompanied by the higher antioxidant capacity in the fungus. In addition to phenolic, flavonoids and tannin compounds, there are several compounds that contribute to the antioxidant activity of filamentous fungi, such as ascorbic acid, β-carotene and tocopherols (α, β and γ) [9]. In the fungus *A. charticola*, flavonoids seemed not to make a significant contribution to the antioxidant activity of the fungus. On the other hand, tannins and other phenolic compounds (other than flavonoids) as well as other compounds associated with antioxidant activity (mentioned above; not quantified in this study) probably had major contributions to the antioxidant activity of *A. charticola*. This inference was in agreement with Smith *et al.* [9] reporting that the antioxidant capacity of *Monascus purpureus* was not a response to flavonoids, but to total phenolics and tannins instead.

## 5. Conclusions

The filamentous fungi isolated from the Indonesian fermented dried cassava exhibited antioxidant potentials as indicated by their capabilities to scavenge ABTS^+^. *A. charticola* had a higher antioxidant capacity than *R. oryzae*. The antioxidant capacity of *A. charticola* was attributed mainly to its phenolics and tannins contents.
